# Cognitive control affects motor learning through local variations in GABA within the primary motor cortex

**DOI:** 10.1038/s41598-021-97974-1

**Published:** 2021-09-17

**Authors:** Shuki Maruyama, Masaki Fukunaga, Sho K. Sugawara, Yuki H. Hamano, Tetsuya Yamamoto, Norihiro Sadato

**Affiliations:** 1 Division of Cerebral Integration, Department of System Neuroscience, National Institute for Physiological Sciences (NIPS), 38 Nishigonaka, Myodaiji, Okazaki, Aichi 444-8585 Japan; 2 Department of Physiological Sciences, School of Life Science, SOKENDAI (The Graduate University for Advanced Studies), Shonan Village, Hayama, Kanagawa 240-0193 Japan; 3 Japan Society for the Promotion of Science (JSPS), Kojimachi, Chiyoda-ku, Tokyo, 102-0083 Japan; 4 Neural Prosthetics Project, Department of Dementia and Higher Brain Function, Tokyo Metropolitan Institute of Medical Science, Kamikitazawa, Setagaya-ku, Tokyo, 156-8506 Japan

**Keywords:** Motor cortex, Cortex, Short-term memory

## Abstract

The primary motor cortex (M1) is crucial for motor learning; however, its interaction with other brain areas during motor learning remains unclear. We hypothesized that the fronto-parietal execution network (FPN) provides learning-related information critical for the flexible cognitive control that is required for practice. We assessed network-level changes during sequential finger tapping learning under speed pressure by combining magnetic resonance spectroscopy and task and resting-state functional magnetic resonance imaging. There was a motor learning-related increase in preparatory activity in the fronto-parietal regions, including the right M1, overlapping the FPN and sensorimotor network (SMN). Learning-related increases in M1-seeded functional connectivity with the FPN, but not the SMN, were associated with decreased GABA/glutamate ratio in the M1, which were more prominent in the parietal than the frontal region. A decrease in the GABA/glutamate ratio in the right M1 was positively correlated with improvements in task performance (*p* = 0.042). Our findings indicate that motor learning driven by cognitive control is associated with local variations in the GABA/glutamate ratio in the M1 that reflects remote connectivity with the FPN, representing network-level motor sequence learning formations.

## Introduction

Motor learning refers to the changing processes of our interactions with the external world^1^. The primary motor cortex (M1) is crucial in motor learning^2^. A recent neuroimaging study demonstrated that the M1 encodes integrated spatiotemporal information of learned finger sequences^3^. Repetitive transcranial magnetic stimulation of the contralateral M1 in humans immediately after training on a ballistic pinch task disrupts consolidation during the offline period after practice^4^. Similarly, muscimol injection into the contralateral M1 of non-human primates selectively disrupts any sequential learned behaviors^5^. A reduction in cortical inhibitory tone is critical for the induction of learning-related M1 plasticity^6,7^. Using an explicit serial reaction time task (SRTT), Kolasinski et al.^8^ observed a significant reduction in GABA in the M1 during learning. Thus, the M1 is part of the neural substrate of sequence learning (i.e., the engram, a persistent change in the brain by a specific experience)^9^. The engram is activated through interactions with retrieval cues (ecphory) and exists between the two active encoding and retrieval processes in a dormant state when the synaptic connection’s strength is stabilized.

Engram formation is not necessarily localized in the single focus of the M1, but may represent the inter-regional network, which is supported by previous studies comparing different training modes of the sequential finger tapping task (i.e., speed-pressure task and SRTT)^10,11^. The speed-pressure task requires participants to practice the given sequence “as fast and as accurately as possible.” This procedure maintains task difficulty because increased speed is directly related to the difficulty level^12–16^. Increasing the difficulty is critical in learning because the degree of challenge indicates the discrepancy between the action plan and the feedback of the sensory outcome of the practice action, which provides the information to update the plan or engram^17^. In addition to motor control, this type of practice for motor learning with increasing difficulty requires flexible cognitive control^18^ of externally and internally directed attention toward the feedback and action planning, respectively. Learning occurs during the sequential finger tapping without speed pressure through the SRTT^8,19–24^, and these training procedures may cause motor learning among different pathways. Hamano et al.^10^ found that SRTT formed dormant state engram in the M1 and dorsal premotor cortex (PMd). In contrast, the speed-pressure task creates an engram in the left parietal regions and the M1, reflecting the updates of the internal model for the speed of execution^10,11^. Considering the M1 is the last component implicated in movement, the internal model for execution speed may be integrated into the M1 creating the motor engram formation at the network level.

Combining magnetic resonance spectroscopy (MRS) and resting-state functional magnetic resonance imaging (fMRI) has allowed for the exploration of the relationship between learning-related changes in the resting-state network and the formation of motor engrams in the M1. A learning-related reduction in GABA levels in the M1 has been found to be correlated with functional connectivity strength changes in the resting-state sensorimotor network (SMN) in long-term motor learning^25^. Baseline GABA levels in the M1 are positively correlated with motor learning-related changes in resting-state functional connectivity between the bilateral M1s and between the right M1 and left superior parietal cortex^26^. Previous studies have evaluated the relationship between GABA levels of the M1 and network changes in motor task-relevant regions. A learning-related reduction in GABA levels associated with functional connectivity may indicate cortical disinhibition, thereby achieving increases in M1 excitability to reconnect with remote areas to encode new skills as network representation; however, the interaction of the M1 with other brain areas during motor learning for engram formation remains unclear.

In the present study, we hypothesized that the fronto-parietal execution network (FPN) provides learning-related information critical for the flexible cognitive control required for practice. To depict how difficulty of speed pressure affects performance, we evaluated the connectivity between the M1 and the executive network cognitively controlling the difficulty of the sequential finger tapping task with speed-pressure combined with MRS measurements of the M1. We simultaneously measured both GABA and glutamate (Glu)^27^, a key excitatory neurotransmitter, to tease apart whether the learning related changes are driven purely by changes in GABA, Glu, or both.

## Materials and methods

### Participants

A total of 43 healthy, right-handed adult volunteers participated in the present study (7 males and 36 females; mean age of 22.9 ± 4.4 years). Handedness was assessed using the Edinburgh Handedness Inventory^28^. None of the participants had a history of neurological or psychiatric diseases. All participants provided written informed consent for participation. The study was conducted according to the Declaration of Helsinki and was approved by the Ethical Committee of the National Institute for Physiological Sciences, Japan. Five participants in learning group were excluded from the analysis due to poor data quality of task performance (n = 2) or MR spectra (n = 3) as described below.

### Experimental design

**Figure 1:**
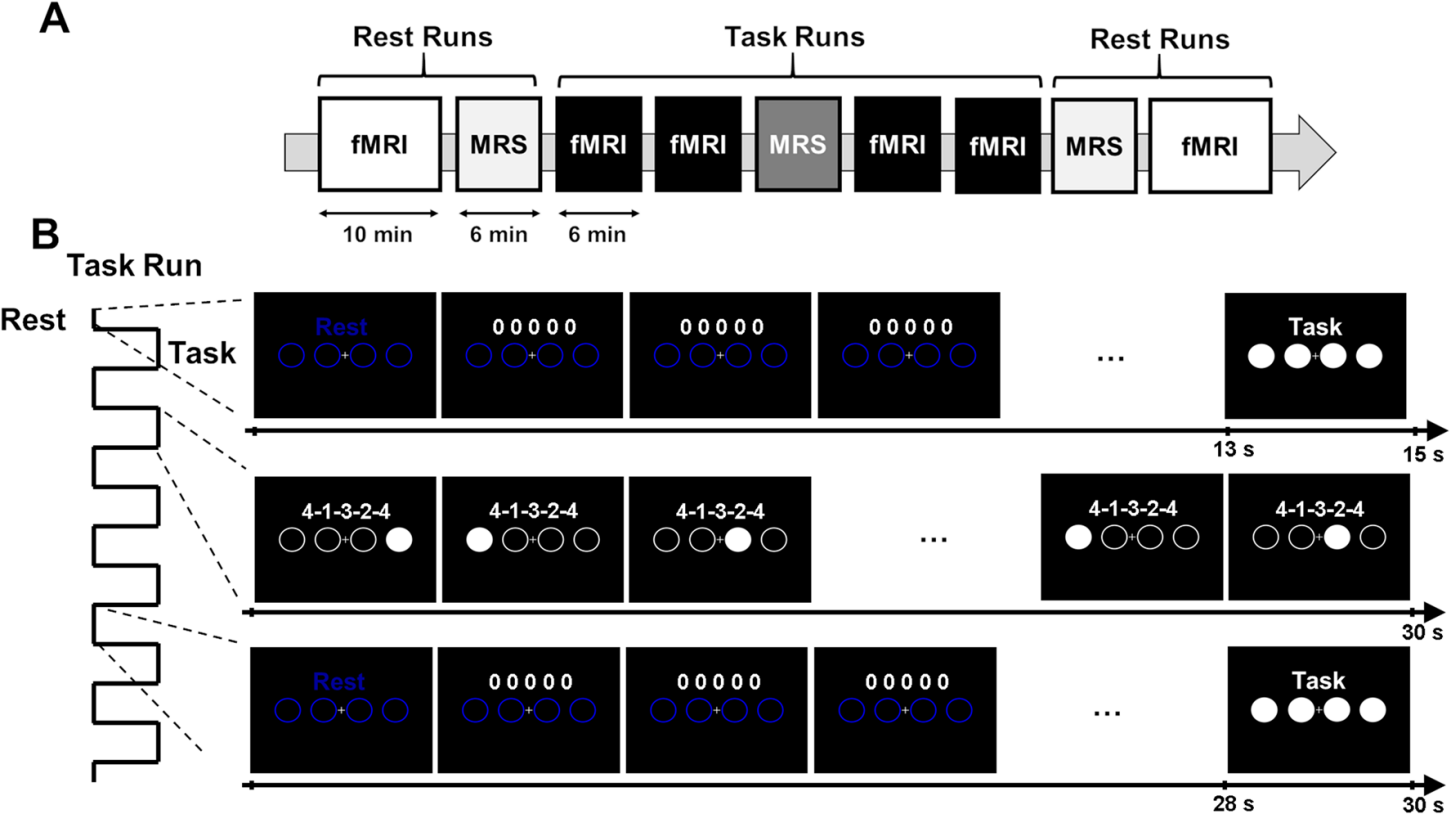
Experimental design. (**A**) The timeline of combined functional magnetic resonance imaging (fMRI) and magnetic resonance spectroscopy (MRS) sessions. The experiment consists of the pre-learning rest session, followed by the task and post-learning rest sessions. During the rest of the sessions, fMRI and MRS scans are conducted before and after motor sequence learning. Meanwhile, during the task session, participants had undergone four fMRI scans and one MRS scan with sequential finger-tapping learning tasks. (**B**) Task design. Each task run (1–5) consists of six cycles of task and rest epochs. Prior to task execution, participants are instructed to retrieve and prepare for the motor sequences following the instructions and closed circles. Participants were indicated using a five-digit sequence (e.g., “4-1-3-2-4”) for 30 s during the task epoch. During the rest epoch, four open blue circles were presented.

We conducted MRS-fMRI experiments using a 7T MRI scanner (MAGNETOM 7T, Siemens Healthineers, Erlangen, Germany) with a 32-channel receiving head coil and a single-channel transmitting coil (Nova Medical Inc., Wilmington, MA, USA). All participants underwent resting-state fMRI and MRS scans before and after the motor sequence learning tasks, as well as one MRS and four fMRI scans during motor sequence learning tasks in the task session (Fig. 1A). Dielectric pads (CaTiO3)^29^ were placed around each participant’s head to improve the B1 transmit field inhomogeneity. All scans were performed within the specific absorption rate limit of the normal operation mode.

### Motor sequence learning task

Thirty participants were asked to perform pre-determined five-digit sequences (“4-1-3-2-4” [n = 17] or “2-3-1-4-2” [n = 13]) as quickly and accurately as possible in the scanner (Fig. 1B)^10,12,13^. Additionally, 13 participants were asked to perform 120 different sequences to assess the non-specific learning as the control condition. The sequence “4-1-3-2-4” corresponds to “index-little-middle-ring-index” fingers. The motor sequence task consisted of six 30 s tapping epochs followed by 30 s rest epochs that were repeated five times (Fig. 1B). The visual feedback signals were displayed using a projector (Optoma EH503; Optoma Inc., Fremont, CA, USA) with a lens (APO 50–500 mm F4.5–6.3 DG OS HSM; SIGMA, Kanagawa, Japan) on a screen viewed by the participants via a mirror mounted to the receiving head coil. Response time was measured using Presentation software (version 16.4; Neurobehavioral Systems, Berkeley, CA, USA). The rest epoch started with the appearance of the instruction “Rest” on the screen for 500 ms, followed by a 500 ms presentation of four blue circles aligned within an equally spaced horizontal array. The instruction “Task” appeared for 2 s at the end of the rest epoch as a signal to the participants to retrieve motor sequences and prepare for their execution (Fig. 1B). The task epoch began with four closed white circles presented for 500 ms, which changed into open circles. During the task epoch, participants tapped the button box (Current Designs, Philadelphia, PA, USA) according to the sequence shown at the top of the screen (i.e., “4-1-3-2-4”). Visual feedback of correct tapping was provided by filling the white circle corresponding to the tapped finger. When the participant provided an incorrect response, the visual feedback signal remained at the previous position until the correct button was tapped. Task performance was measured using transition time (TT), defined as the average time between two correct button responses per epoch. The performance improvement was calculated using the following equation:

$$ Performance\; improvement \left( \% \right) = \frac{{\left( {TT_{1} {-}TT_{5} } \right)}}{{TT_{5} }} \times 100 $$

where TT_1_ indicates the transition time at run 1 and TT_5_ indicates the transition time at run 5. The task performance data were analyzed using repeated-measures ANOVA, with run as a factor, performed using SPSS (version 25; IBM, Armonk, NY, USA). Two participants were excluded due to a statistical outlier in the TT values (> 2 standard deviations [SD]).

### Structural data acquisition

Three dimensional T1-weighted (T1w) images were acquired for anatomical reference (Magnetization Prepared Rapid Acquisition Gradient Echo [MPRAGE]^30^, repetition time [TR]/TE = 3000/3.08 ms; inversion time [TI] = 1200 ms; field of view = 240 × 225 mm^2^; matrix size = 320 × 320; slice thickness = 0.75 mm; 224 slices; generalized auto-calibrating partially parallel acquisitions [GRAPPA]^31^ acceleration factor = 3; bandwidth = 230 Hz/Px; flip angle = 14°; acquisition time = 4 min 50 s).

### fMRI data acquisition

fMRI images were acquired before, during, and after the motor sequence learning tasks using a multiband gradient-echo echo-planar imaging sequence^32^. The scan parameters were set as per the Human Connectome Project (HCP) 7T protocol (TR/TE = 1000/22.2 ms; field of view = 208 × 208 mm^2^; matrix size = 130 × 130; slice thickness = 1.6 mm; 85 slices; multi-band/GRAPPA acceleration factor = 5/2; bandwidth = 1924 Hz/Px; flip angle = 45°)^33^. The spin echo field map was acquired (TR/TE = 3000/60 ms; field of view = 208 × 208 mm^2^; matrix size = 130 × 130; slice thickness = 1.6 mm; 85 slices; multi-band/GRAPPA acceleration factor = 5/2; bandwidth = 1924 Hz/Px; flip angle = 180°; acquisition time = 1 min 26 s)^34^. A B1 transmit field map in the center of the brain, around the slice of the M1 hand knob area, was acquired for each participant to optimize the input power for accurately producing a 90° pulse for all fMRI scans. In particular, participants were instructed to keep their eyes open while viewing a fixation cross and to avoid having any specific thoughts or falling asleep during resting-state fMRI scans.

### MRS data acquisition

**Figure 2:**
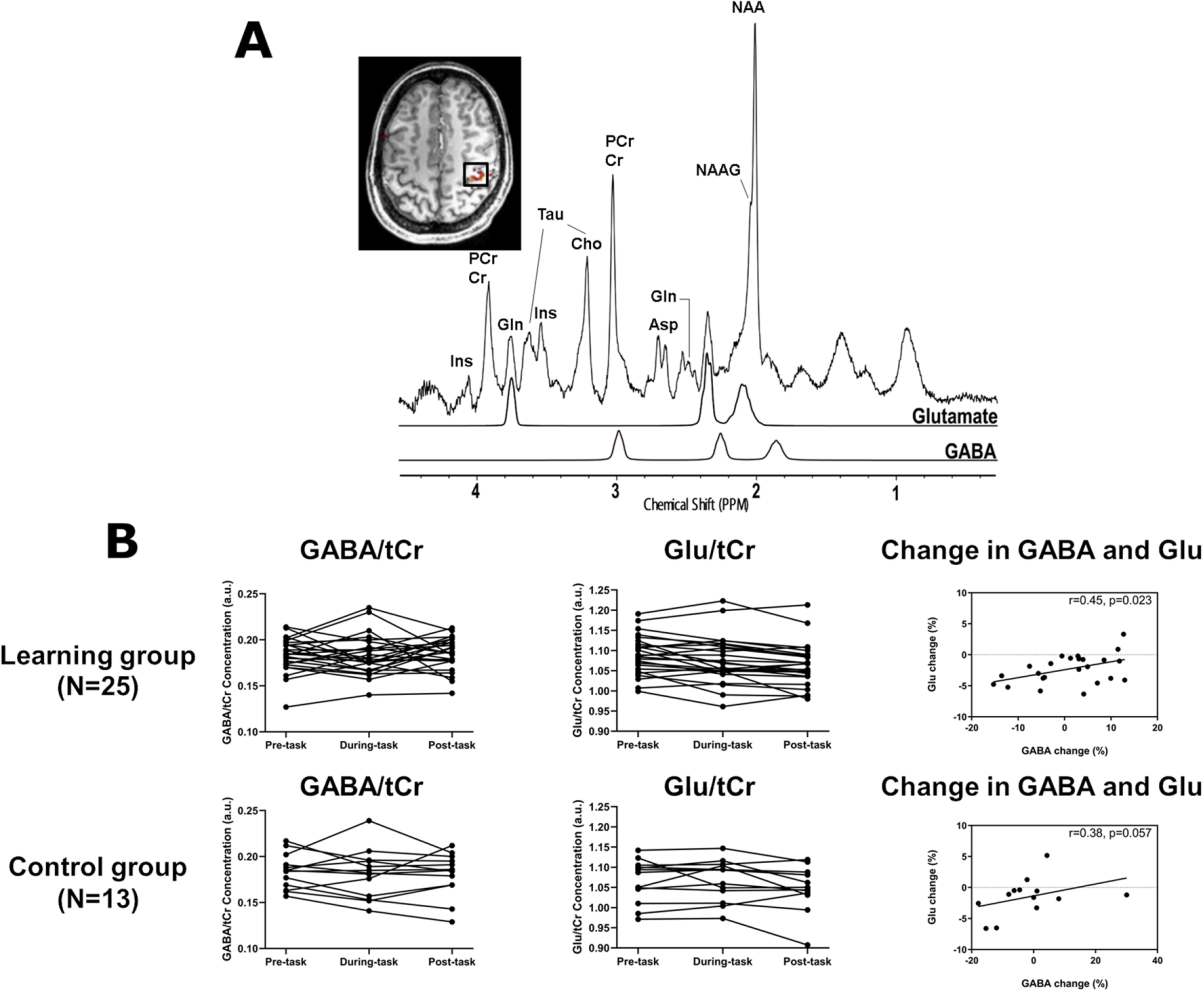
Magnetic resonance spectra and neurotransmitter levels. (**A**) MR spectra of the right primary motor cortex (M1) acquired in one subject (STEAM, TR/TE = 5000/5.68 ms; mixing time = 40 ms; vector size = 2048; bandwidth = 4000 Hz/Px; average = 64), as well as LCModel spectra fit for Glutamate and GABA are shown. The 2 × 2 × 2 cm^3^ volume of interest (black square) is centered over the hand knob area of M1 identified using fMRI during a finger opposition task (red) and is superimposed on the T1w MPRAGE image. Cho, choline; Cr, creatine; PCr, phosphocreatine; Gln, glutamine; Glutamate, Ins, myo-inositol; NAA, N-acetylaspartate; NAAG, N-acetylaspartylglutamate; Asp, aspartateaspartate; Tau, taurine. (**B**) Task-related changes in GABA and Glutamate (Glu) levels. The left and middle columns indicate the concentrations of GABA and Glu within the right M1 during the pre-task, during-task, and post-task of Learning Group (Top) and those of Control Group (Bottom). The right column show the association between GABA and Glu changes post motor sequence learning task of the Learning Group (Top) and Control Groups (Bottom).

A 2 × 2 × 2 cm^3^ volume of interest was centered over the right M1 hand knob area (Fig. 2A), without dura, on T1w MPRAGE images. The hand knob area was identified using fMRI during a sequential finger opposition task with the left hand (TR/TE = 1000/24 ms; field of view = 192 × 192 mm^2^; matrix = 96 × 96; slice thickness = 2 mm; 20 slices; GRAPPA acceleration factor = 2; bandwidth = 2170 Hz/Px; flip angle = 45°; acquisition time = 3 min 30 s). The localization task did not affect our current data because there was no component of learning of the sequence, which was our main focus. Ultra-short TE MRS data were acquired before, during, and after the motor sequence learning task using the STEAM sequence (TR/TE = 5000/5.68 ms; mixing time = 40 ms; vector size = 2048; bandwidth = 4000 Hz/Px; average = 64) with VAriable Power RF pulses with Optimized Relaxation delays (VAPOR) water suppression^35,36^. The STEAM sequence was combined with outer volume suppression to improve localization performance. A 4-s average water reference signal was acquired for eddy current correction and absolute quantification of the metabolites. Before data acquisition, all first- and second-order shim terms were automatically adjusted with the fast automatic shim technique using echo-planar signal readout for mapping along with projections^37,38^. In addition, B1 transmit field strength for localization pulses, and VAPOR water suppression was adjusted for individual participants.

### HCP-style structural data acquisition with 3T MRI and preprocessing

In addition to the MRS-fMRI data acquisition using 7  MRI, the HCP-style structural data of all participants were obtained using a 3T MRI scanner (Magnetom Verio, Siemens Healthcare, Erlangen, Germany) with a 32-channel receiving head coil (Siemens Healthcare, Erlangen, Germany). The obtained 3T MRI data were utilized to correct the geometric distortion of the 7T MR data (see below, *fMRI preprocessing*)^39^. Scan parameters were as per the HCP 3T protocol with minor modifications^40^. Three-dimensional T1w images were acquired (MPRAGE^30^, TR/TI/TE = 2400/1060/2.24 ms; field of view = 256 × 240 mm^2^; matrix size = 320 × 320; slice thickness = 0.8 mm; 224 slices; GRAPPA acceleration factor = 2; bandwidth = 210 Hz/Px; flip angle = 8°; acquisition time = 6 min 38 s; measurement = 2). Three-dimensional T2 weighted (T2w) images were acquired (Sampling Perfection with Application optimized Contrast using different angle Evolutions [SPACE]^41^, TR/TE = 3200/560 ms; field of view = 256 × 240 mm^2^; matrix size = 320 × 320; slice thickness = 0.8 mm; 224 slices; GRAPPA acceleration factor = 2; bandwidth = 744 Hz/Px; turbo factor = 167; acquisition time = 6 min; measurement = 2). All data were processed using the structural pipeline (PreFreeSurfer, FreeSurfer, and PostFreeSurfer) of the minimal HCP preprocessing pipeline version 4.0.0-alpha.5, including the following steps: gradient magnetic field nonlinearity distortion correction, T2w images to T1w image registration, and Montreal Neurologic Institute (MNI) volume registration^40^.

### MRS data analysis

Raw MRS data were post-processed using the MATLAB (version R2018a) toolbox MRspa version 1.5f. (https://www.cmrr.umn.edu/downloads/mrspa/). Motion-corrupted data were removed to improve the spectral quality. To quantify the proportion of gray matter (GM), white matter (WM), and cerebrospinal fluid (CSF) fractions in the volume of interest, segmentation in SPM (version 12) was applied to the T1w MPRAGE images. All neurotransmitter concentrations were corrected for GM and WM concentrations, as previously described^42^. Eddy current and frequency corrections were performed using a water reference scan, and the zero- and first-order phases of the array coil were aligned using the cross-correlation method of MRspa. Subsequently, LCModel (version 6.3-1N; Stephen Provencher, Inc., Oakville, ON, Canada) was used to quantify the concentration of neurochemicals within the chemical shift range of 0.5–4.1 ppm^43,44^. Other parameters in the LCModel were as reported previously^45^. The metabolites were estimated with a simulated basis set of 7T STEAM sequence provided by LCModel. The basis set includes the spectra for the following metabolites: alanine, aspartate (Asp), Cr, phosphocreatine (PCr), GABA, glucose, glutamine (Gln), Glu, glycerophosphocholine, phosphocholine, choline (Cho), myo-inositol (Ins), N-acetyl aspartate (NAA), N-acetyl aspartylglutamate (NAAG), scyllo-inositol, taurine (Tau), and macromolecules. The concentrations of GABA and Glu were normalized to that of tCr, which is known to be a high and stable concentration in the human brain^27^. We found significant positive correlation between learning related change in GABA and Glu (Fig. 2B). As GABA is generated from Glu^27^, we calculated the change in the GABA/Glu ratio after the motor sequence learning task using the following equation:

$$ \frac{GABA}{{Glu}}change \left( \% \right) = \frac{{\left( {\frac{GABA}{{Glu_{post} }} - \frac{GABA}{{Glu_{pre} }}} \right)}}{{GABA/Glu_{pre} }} \times 100 $$

where GABA/Glu_pre_ and GABA/Glu_post_ indicate the GABA/Glu ratio at pre- and post-task, respectively. Two-way repeated-measures ANOVA was conducted using SPSS, with the concentrations of GABA and Glu at different time points (pre-, during-, and post-task) as a factor. The CRLB, water linewidth at FWHM, and signal-to-noise ratio (SNR) were used for the quality control of spectra^44^. The CRLB and SNR were calculated using LCModel, and water linewidth was obtained by fitting to the additional water spectrum using MATLAB. Data were excluded when CRLB > 15% (n = 2), linewidth > 19 Hz (n = 1), or SNR < 30. Two-way repeated-measures ANOVA was performed on the CRLB, water linewidth, and SNR with the experimental group (learning vs. movement) as a between-subjects factor and time points (pre-, during-, and post-task) as a within-subjects factor, using SPSS.

### fMRI preprocessing

All fMRI data were processed using the functional pipeline (fMRIVolume) of the minimal HCP preprocessing pipeline^39^. This pipeline included the following steps: motion correction, gradient magnetic field nonlinearity distortion correction, field map-based distortion correction (Topup)^34^, nonlinear registration into 3T MNI structure data, and grand-mean intensity normalization. Finally, volume-based smoothing with a 5 mm full width at half maximum (FWHM) Gaussian kernel was applied.

### Task-based fMRI data analysis

Task-based fMRI analysis was performed using SPM12 in MATLAB. A general linear model (GLM) was fitted to the fMRI data for each participant^46,47^. The fMRI time series for preparation phases 2 s before task execution and execution phases were modeled with boxcar functions convolved with the canonical hemodynamic response function. Each run consisted of six execution- and preparation-related regressors. The mean design orthogonality between the execution and preparation phases was − 0.0137 ± 0.054, − 0.0141 ± 0.054, − 0.0137 ± 0.054, and − 0.0139 ± 0.054 for run 1, 2, 4, and 5, respectively. Temporal high-pass filtering with a cutoff frequency of 1/128 Hz was applied. Using a first-order autoregressive model, the serial autocorrelation was estimated from the pooled active voxels with the restricted maximum likelihood procedure and subsequently used to whiten the data^48^. Several nuisance covariates, including six head motion parameters and CSF time-series, were incorporated into the model. The parameter estimates for each execution- and preparation-related regressor were assessed using constant and predefined linear contrasts. Increasing contrast vectors were defined numerically as an increment of one per run, maintaining the mean equal to zero.

For group-level analysis of task-based fMRI data, one-sample t-tests of participants’ contrast images were performed^49^. The resulting set of voxel values for each contrast constituted the SPM{t}. We calculated the T-score of linear increments in preparation-related activity in the right M1 in non-specific learning. The statistical threshold was set at *p* < 0.05, FWE-corrected at the voxel-level^50^, unless otherwise specified.

### Anatomical labeling and visualization

MRIcron (https://www.nitrc.org/projects/mricron) was used to display fMRI activation maps on a standard brain image. The Automated Anatomical Labeling atlas was used for anatomical labeling^51^.

### Resting-state fMRI data analysis

Resting-state functional connectivity analysis was conducted using the CONN toolbox (version 17; https://web.conn-toolbox.org/)^52^. An anatomical component-based noise correction method (aCompCor)^53^ was applied to remove the five components of signals from WM, CSF, and residual head motion-related signals through linear regression. A temporal bandpass filtering of 0.008–0.090 Hz was applied.

Seed-to-voxel correlation analysis was performed at the individual level. We selected the preparation-related increased voxels in the M1 (MNI: x = 36, y =  − 25, z = 51), determined by the second-level analysis of task-based fMRI (FWE voxel-level corrected *p* < 0.05), as a seed region of interest (ROI; Fig. 6). An individual seed-based functional connectivity map was obtained by computing Pearson’s correlation coefficients between the time-series from the M1 seed ROI and the time-series of all other voxels across the whole brain. Fisher’s r-to-*z* transformation was used to convert the correlation coefficients into *z-*scores. M1-seeded functional connectivity changes were integrated using the following equation in AFNI (version 18.1.32; https://afni.nimh.nih.gov/):

$$ Connectivity \;change = \mathop \sum \limits_{{}}^{{}} \left( {Connectivity_{post} {-} Connectivity_{pre} } \right) $$

where Connectivity_pre_ and Connectivity_post_ are the pre- and post-task functional connectivity values, respectively.

We calculated the changes in functional connectivity within ROIs of the SMN and FPN defined from CONN’s ICA analyses of the HCP dataset of 497 individuals. The SMN includes the supplementary motor cortex and bilateral sensorimotor cortex, whereas the FPN consists of the bilateral LPFC and PPC. The correlations between GABA/Glu changes within the M1 and M1 seed-based functional connectivity changes were analyzed using linear regression analysis.

## Results

### MRS spectra

Figure 2A represents an example of MR spectra within the M1 obtained using the 7T MR system. To assess whether the changes in metabolite concentrations were due to fluctuations in spectral quality, we evaluated the Cramer–Rao lower bounds (CRLB), linewidth, and signal-to-noise ratio (SNR). MRS spectra provided reliable estimates of GABA, Glu, and total creatine (tCr), with a CRLB < 15%. Two-way repeated-measures analysis of variance (ANOVA) revealed no significant main effect of time (pre-task vs. during-task vs. post-task) or group (learning vs. movement) on CRLB, linewidth, or SNR (Table 1).

**Table 1:** Magnetic resonance spectroscopy spectra quality of pre-task, during-task, and post-task periods.

	CRLB (GABA)	CRLB (Glu)	CRLB (tCr)	Linewidth (water)	SNR	tCr (mM)
**Learning group**						
Pre-task	9.8 ± 1.2	2.0 ± 0.2	2.0 ± 0.2	12.8 ± 0.9	42.5 ± 2.6	10.3 ± 0.6
During-task	9.4 ± 1.2	2.0 ± 0.2	2.0	12.7 ± 1.0	42.2 ± 2.3	10.3 ± 0.6
Post-task	9.6 ± 0.8	2.1 ± 0.3	2.0 ± 0.2	12.8 ± 1.0	41.6 ± 2.7	10.3 ± 0.6
**Control group**						
Pre-task	9.5 ± 1.0	2.1 ± 0.3	1.9 ± 0.3	12.4 ± 1.3	43.8 ± 3.5	10.7 ± 0.6
During-task	9.5 ± 1.0	2.1 ± 0.4	1.9 ± 0.3	12.7 ± 1.8	42.5 ± 4.4	10.5 ± 0.6
Post-task	9.9 ± 1.2	2.1 ± 0.3	1.9 ± 0.3	12.7 ± 1.7	43.2 ± 3.5	10.7 ± 0.6
Time × group	*F*[2, 72] = 1.295 *p* = 0.2801	*F*[2, 72] = 1.194 *p* = 0.310	*F*[2, 72] = 0.251 *p* = 0.778	*F*[2, 72] = 3.008 *p* = 0.056	*F*[2, 72] = 2.008 *p* = 0.142	*F*[2, 72] = 2.879 *p* = 0.066
Main effect of time	*F*[2, 72] = 0.985 *p* = 0.378	*F*[2, 72] = 0.398 *p* = 0.664	*F*[2, 72] = 0.251 *p* = 0.723	*F*[2, 72] = 1.213 *p* = 0.299	*F*[2, 72] = 3.076 *p* = 0.054	*F*[2, 72] = 1.842 *p* = 0.166
Main effect of group	*F*[1, 36] = 0.074 *p* = 0.787	*F*[1, 36] = 0.242 *p* = 0.626	*F*[1, 36] = 3.121 *p* = 0.411	*F*[1, 36] = 0.121 *p* = 0.730	*F*[1, 36] = 1.207 *p* = 0.279	*F*[1, 36] = 3.613 *p* = 0.065

Two-way repeated-measures ANOVA analysis was applied to the CRLB, water linewidth, and SNR separately, with the experimental group (learning vs. control) as a between-subjects factor while time points (pre-task, during-task, and post-task) as a within-subjects factor using SPSS. Data are presented as mean ± standard deviation (SD).

*CRLB* Cramer–Rao lower bounds, *Glu* glutamate, *tCr* total creatine, *SNR* signal-to-noise ratio.

Figure 2B presents the distribution of the concentrations of GABA/tCr and Glu/tCr in the pre-, during-, and post-task periods and the relationship between GABA and Glu changes. The variation in neurotransmitter concentration was analyzed using repeated-measures ANOVA with time (pre-task vs. during-task vs. post-task) and group (learning vs. control) as factors. No significant main effect of time (*F*_(2,72)_ = 0.144; *p* = 0.856) or group (*F*_(1,36)_ = 0.107; *p* = 0.754) on GABA/tCr concentration was observed. Although a significant main effect of time (*F*_(2,72)_ = 10.72; *p* = 8.410 × 10^–5^) on Glu/tCr was observed, no significant main effect of group (*F*_(1,36)_ = 0.718; *p* = 0.402) or any interaction effects (*F*_(2,72)_ = 1.380; *p* = 0.258) were found. Post-hoc tests revealed that Glu/tCr decreased significantly from pre- to post-task (*p* < 0.001, Sidak correction for multiple comparisons) and during- to post-task (*p* = 0.003) but was not significant from pre- to during-task (*p* = 0.642). Moreover, changes in Glu and GABA from pre- to post-task were significantly correlated (*r*_(25)_ = 0.45, *p* = 0.023). Through the analysis of covariance (ANCOVA), changes in Glu were included as the dependent variable, and GABA changes and group were independent variables, and we found that changes in GABA were significant (*F*_(1,34)_ = 7.503, *p* = 0.010), but not for the main effect of group (*F*_(1,34)_ = 1.677, *p* = 0.204) or their interaction (*F*_(1,34)_ = 0.054, *p* = 0.818).

### Task performance

**Figure 3:**
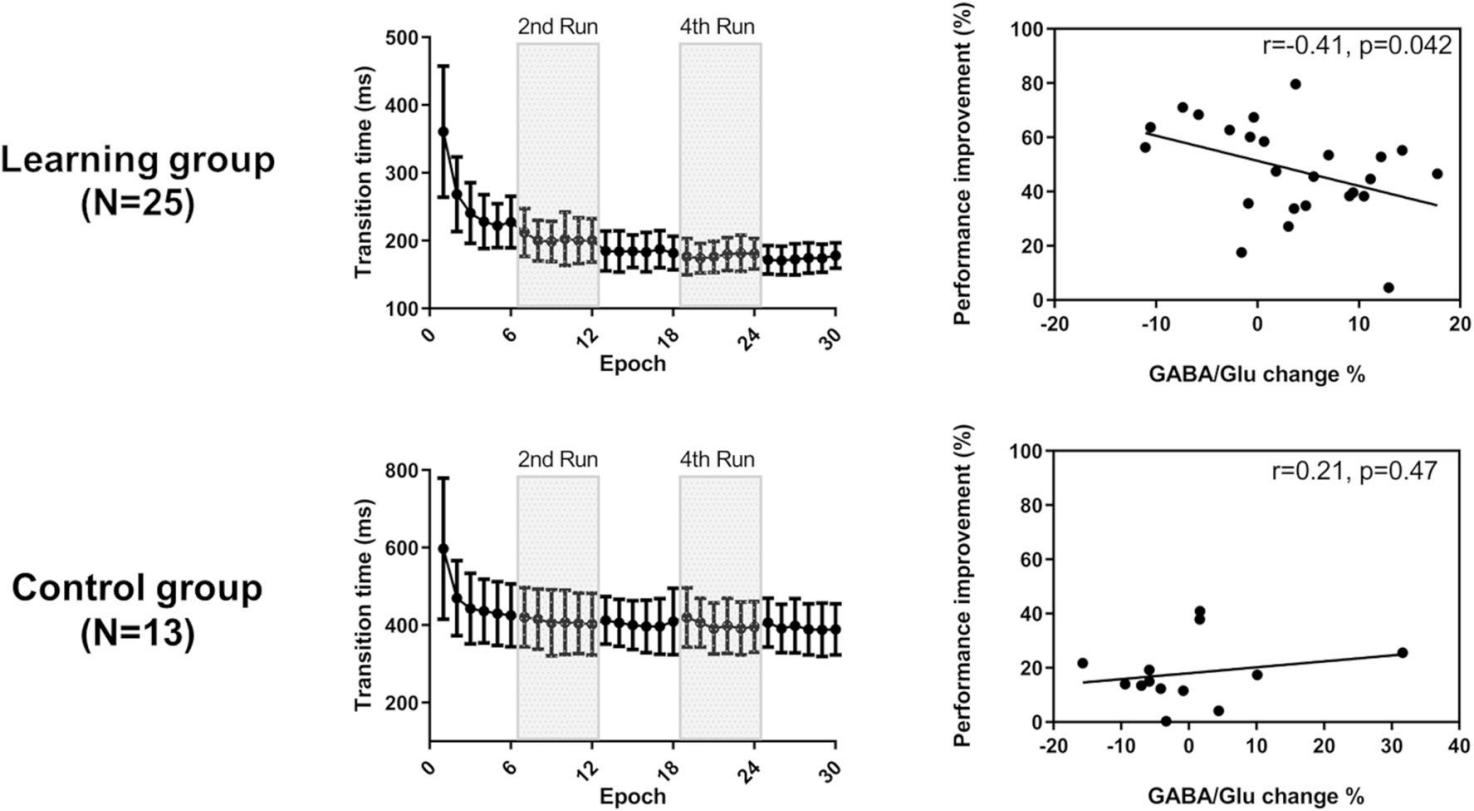
Changes in GABA/Glu ratio with behavioral performance improvement. Task performance change in the motor sequence learning (n = 25, Top Left) and control groups (n = 13, Bottom Left). Task performance was measured using transition time defined as the median time between two correct button responses per epoch. Data are presented as mean ± standard deviation (SD). The right column show the association between GABA/Glu changes within the right primary motor cortex and performance improvement in the learning (Top Right) and control groups (Bottom, Right).

Task performance was evaluated using the transition time of the consecutive finger tapping (Fig. 3). The mean transition times (± standard deviation, in ms) in the learning group were 258.063 ± 46.213, 202.490 ± 31.049, 184.320 ± 26.285, 178.237 ± 22.600, and 173.673 ± 19.417 for runs 1–5, respectively. In the control group, the mean transition times (± standard deviation, in ms) were 466.960 ± 96.564, 409.300 ± 77.179, 403.520 ± 64.867, 400.546 ± 66.247, and 393.746 ± 64.553 for runs 1–5, respectively. The performance improvement was analyzed using repeated-measures ANOVA with time (Runs 1–5) and group (learning vs. control) as factors. We found significant main effects of time (*F*_(4,144)_ = 102.700; *p* = 4.347 × 10^−12^) and group (*F*_(1,36)_ = 177.800; *p* = 1.705 × 10^−15^), but no significant interaction effect was found (*F*_(4,144)_ = 1.250; *p* = 0.293).

Post-hoc one-sample t-tests revealed that the transition time did not significantly differ between runs 4 and 5 (*p* = 0.389, Bonferroni corrected) in the learning group, indicating that performance plateaued. A significant difference was found in all pairs except between runs 4 and 5 in the learning group. Alternatively, a significant difference was found between runs 1 and 5 in the control group.

Next, the relationship between the changes in GABA/Glu ratio within the M1 and performance improvements in the learning and control groups was evaluated. A negative correlation was observed between the change in the GABA/Glu ratio and performance improvement (*r*_(25)_ =  − 0.41, *p* = 0.042; Fig. 3, top right). Using an ANCOVA, with performance improvement as the dependent variable and GABA/Glu change and group as independent variables, we found a significant effect of group (*F*_(1,34)_ = 39.423, *p* < 0.001) and no main effect of GABA/Glu (*F*_(1,34)_ = 2.371, *p* = 0.133); however, their interaction was trending toward significance (*F*_(1,34)_ = 3.423, *p* = 0.073).

### Execution- and preparation-related activities

**Figure 4:**
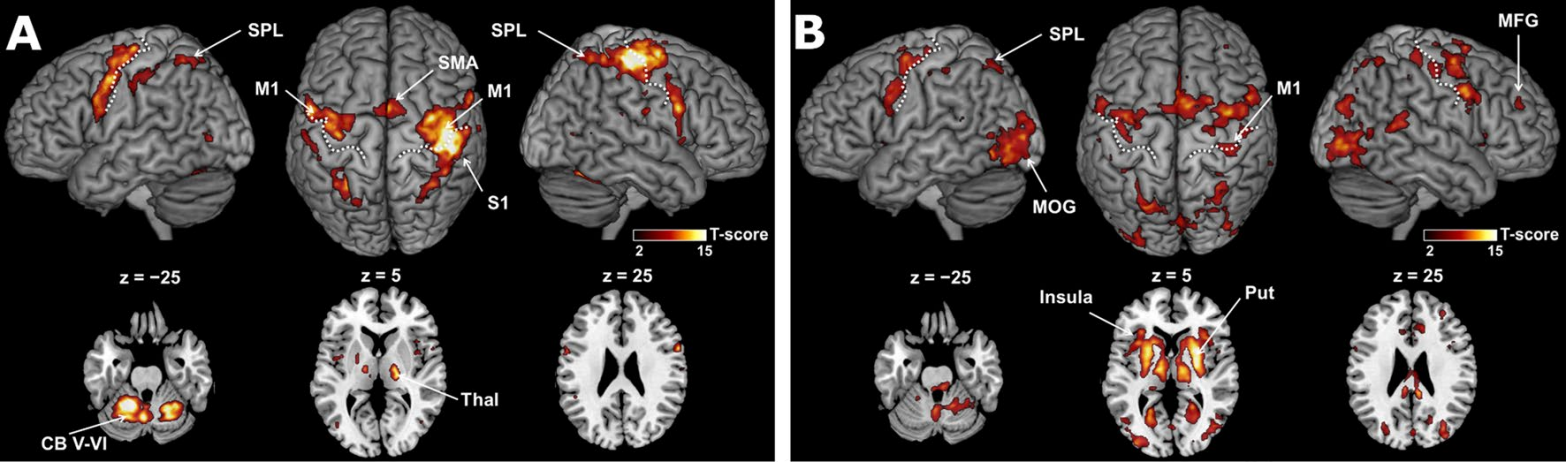
Constant changes during execution and preparation. (**A**) Execution-related and (**B**) preparation-related activity superimposed on the surface-rendered high-resolution magnetic resonance imaging of the template brain. The white dotted lines delineate the central sulcus. The level of statistical significance is set to *p* < 0.05, family-wise error corrected for multiple comparisons at the voxel-level. CB, cerebellum; SPL, superior parietal lobule; MFG, middle frontal gyrus; MOG, middle occipital gyrus; S1, primary somatosensory cortex; M1, primary motor cortex; Thal, thalamus; Put, putamen; SMA, supplementary motor area.

Task-based fMRI analysis revealed the task execution-related activity in the bilateral M1, cerebellum (CB) lobules, supplementary motor area (SMA), thalamus (Thal), superior parietal lobule (SPL), and right primary somatosensory cortex (S1; voxel-level family-wise error [FWE]-corrected *p* < 0.05; Fig. 4A). The preparation-related activity was observed in the bilateral putamen (Put), insula, M1, SMA, Thal, SPL, middle occipital lobe, right S1, and middle frontal gyrus (MFG; voxel-level FWE-corrected *p* < 0.05; Fig. 4B).

### Linear increments in execution- and preparation-related activities

**Figure 5:**
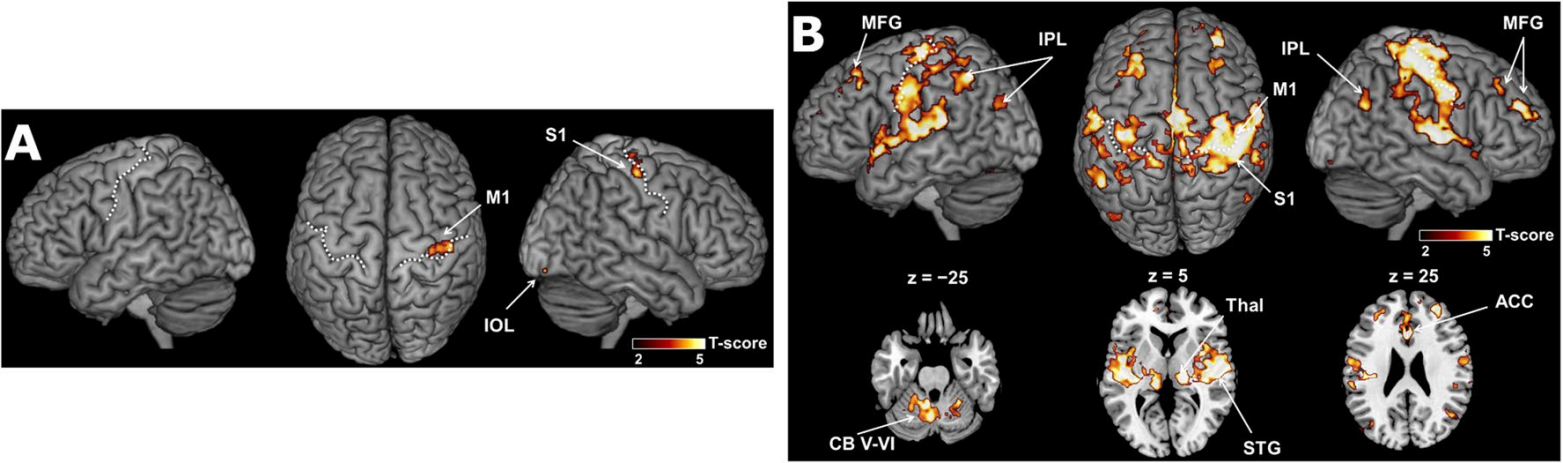
Learning related changes during execution and preparation. Linear increments in (**A**) execution-related and (**B**) preparation-related activity superimposed on the surface-rendered high-resolution magnetic resonance imaging of the template brain. The white dotted lines indicate the central sulcus. The level of statistical significance is set to *p* < 0.05, family-wise error corrected for multiple comparisons at the cluster-level. CB, cerebellum; IPL, inferior parietal lobule; MFG, middle frontal gyrus; S1, primary somatosensory cortex; M1, primary motor cortex; SMA, supplementary motor area; STG, superior temporal gyrus; Thal, thalamus; ACC, anterior cingulate cortex.

We observed linear increments in execution-related activity in the right M1, S1, and inferior occipital lobe with a lenient threshold (uncorrected *p* < 0.001 at the voxel-level and FWE-corrected *p* < 0.05 at the cluster level; Fig. 5A). In contrast, linear increments in preparation-related activity were observed in the right M1 and S1, and SMA. A linear increase in preparatory activity was also found in fronto-parietal regions, including the bilateral inferior parietal lobule (IPL), MFG, superior temporal gyrus, Thal, CB lobules, anterior cingulate cortex, and middle cingulate cortex (uncorrected *p* < 0.001 at the voxel-level for height threshold; FWE-corrected *p* < 0.05 at the cluster level; Fig. 5B).

### Resting-state functional connectivity before and after motor learning

**Figure 6:**
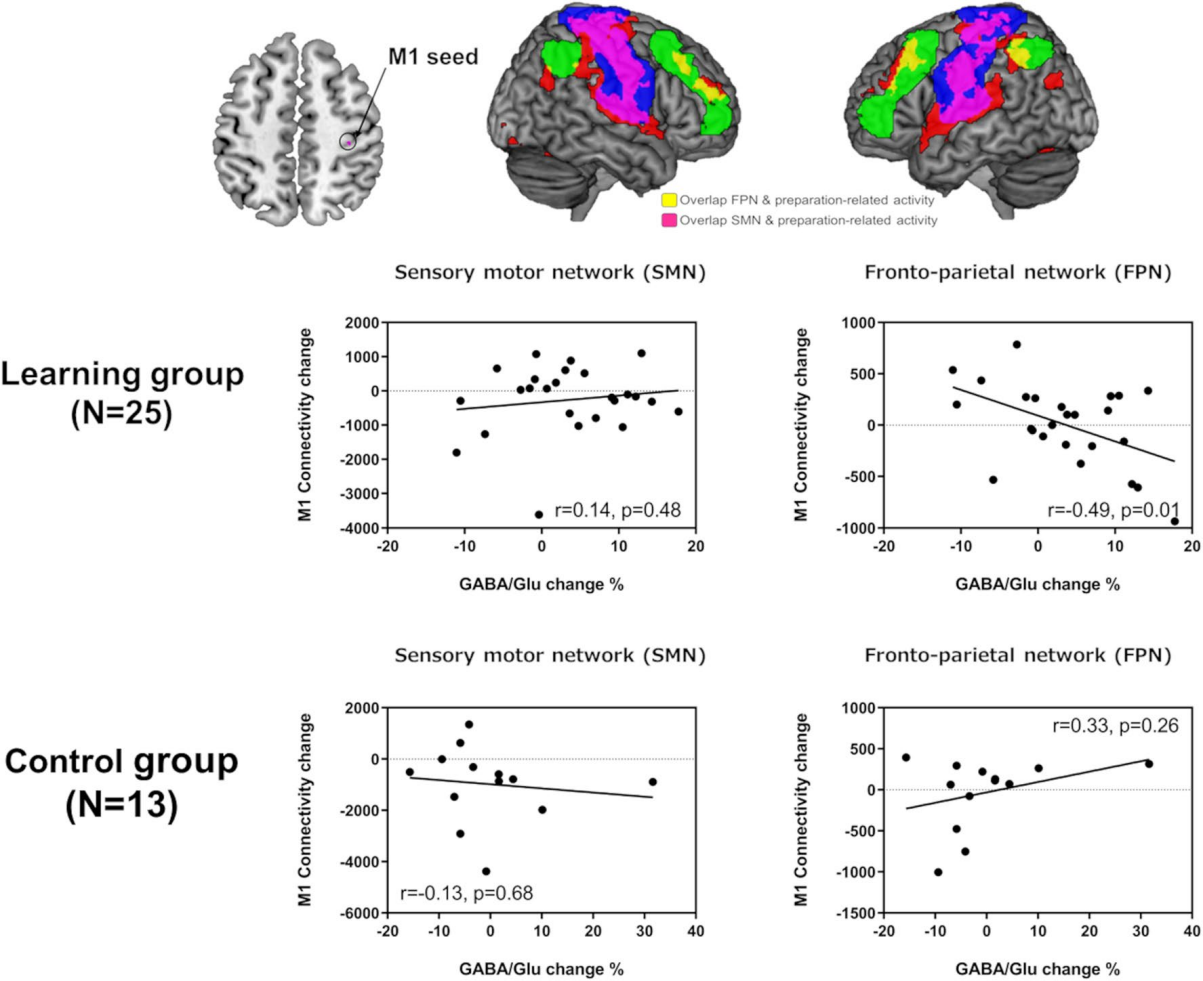
Region of interest (ROI)-based analysis of functional connectivity between the right primary motor cortex (M1) and sensorimotor network (SMN), and fronto-parietal network (FPN). (Top Left) M1 seed ROI depicted by the linear increments in preparation-related activity of the Learning Group. The level of statistical significance is set at p < 0.05, family-wise error corrected for multiple comparisons at the voxel-level. (Top Right) ROI overlap of SMN (blue) and FPN (green) with the learning-related network, depicted by the linear increments in preparation-related activity using task functional magnetic resonance imaging (red). SMN and FPN are defined based on the CONN toolbox. (Middle) Relationships between Glu/GABA changes within the right M1 and M1 seed-based resting-state functional connectivity changes in the SMN (Middle left) and FPN (Middle right) after motor sequence learning of the Learning and Control Groups (Bottom). M1 seed-based resting-state functional connectivity changes were calculated using the sum of changes in the connectivity values between the pre- and post-task periods in the networks.

The learning-related network, depicted as linear increments in preparation-related activity during task-based fMRI, overlapped with the FPN and SMN templates provided by the CONN toolbox (Fig. 6, top right). The relationships between GABA/Glu changes within the M1 and resting-state M1 seed-based functional connectivity changes in the SMN and FPN after learning were investigated. There was a negative correlation between M1 connectivity with the FPN and changes in GABA/Glu within the M1 from pre- to post-task (*r*_(25)_ =  − 0.49, *p* = 0.01). Using an ANCOVA and including connectivity change as a dependent variable and GABA/Glu change and group as independent variables, we found a significant interaction between change in GABA/Glu and group (*F*_(1,34)_ = 7.482, *p* = 0.010) without a significant main effect of group (*F*_(1,34)_ = 0.844, *p* = 0.365) or GABA/Glu change (*F*_(1,34)_ = 0.819, *p* = 0.372). M1 connectivity with the SMN and changes in GABA/Glu within the M1 from pre- to post-task were not correlated (*r*_(25)_ = 0.14, *p* = 0.48) with learning. Using another ANCOVA, with connectivity change as a dependent variable and GABA/Glu change and group as independent variables, we found no significant interaction between GABA/Glu change and group (*F*_(1,34)_ = 0.648, *p* = 0.427), or main effects of group (*F*_(1,34)_ = 2.304, *p* = 0.138) or GABA/Glu change (*F*_(1,34)_ = 0.005, *p* = 0.943).

**Figure 7:**
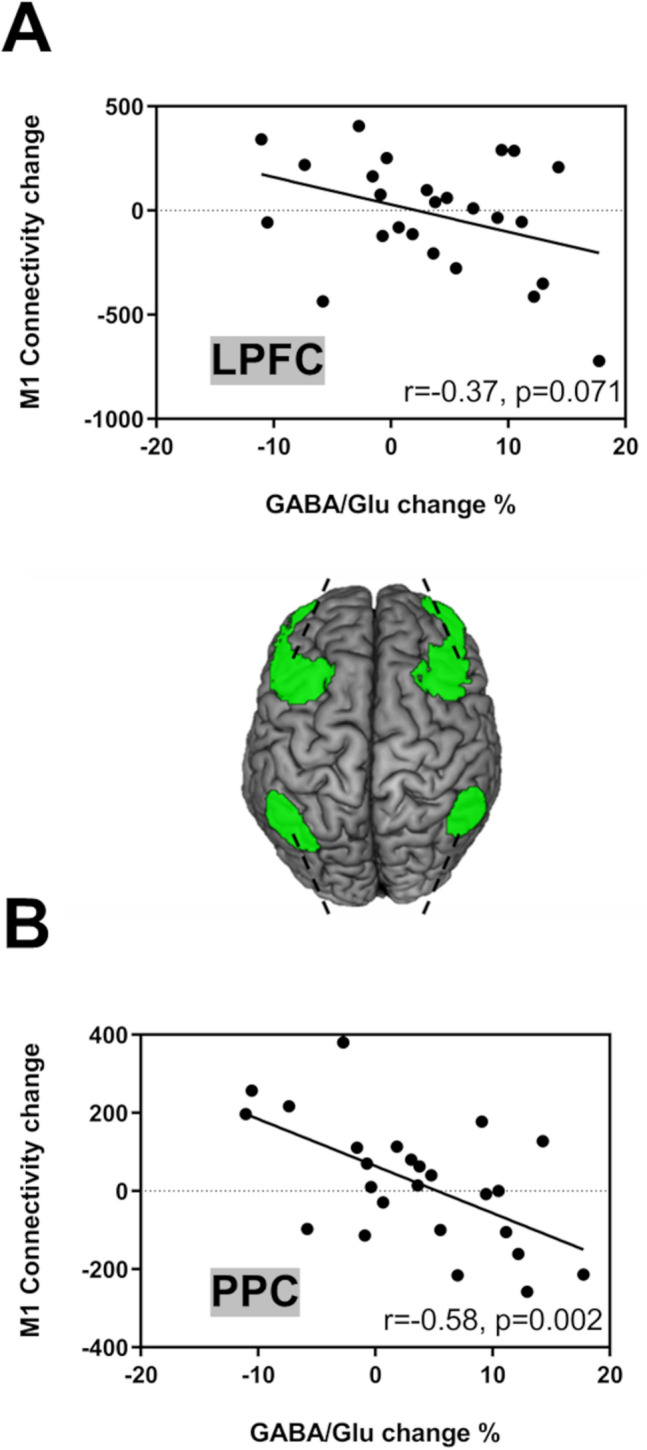
Region of interest-based analysis of functional connectivity between the right primary motor cortex (M1) and subregions of fronto-parietal network (FPN) of Learning Group (n = 25). Associations between the changes in Glu/GABA ratio within the right M1 and M1 seed-based resting-state functional connectivity changes in (**A**) lateral prefrontal cortex (LPFC) and (**B**) posterior parietal cortex (PPC) of the FPN (green) after motor sequence learning.

The correlation between the FPN and changes in the GABA/Glu ratio was more prominent in parietal regions than frontal regions (e.g., lateral prefrontal cortex [LPFC], *r*_(25)_ =  − 0.37, *p* = 0.071; posterior parietal cortex [PPC], *r*_(25)_ =  − 0.58, *p* = 0.002; Fig. 7).

## Discussion

In the present study, we used task-based and resting-state fMRI and MRS to assess network-level changes by motor sequence learning requiring cognitive control. Task-based fMRI revealed learning-related changes in the fronto-parietal regions, including the right M1. We found that GABA/Glu in the M1 reflects its functional connectivity with the FPN and improvements in performance. Thus, the M1 represents a hub of network-level engram formation.

### Behavioral effect of learning

Both learning and control groups underwent five runs of training, each of which contained six task epochs. Both groups demonstrated differing transition times across runs. Post-hoc tests showed that in the control group, only the first run differed from other runs. Furthermore, control group practiced the tapping of different sequences (first-look sequence) throughout the run, thus this learning effect is not specific to the particular sequence as in learning group. For the 13 control participants, the last epoch of the fifth run demonstrated an average of 393.746 ± 64.553 ms. Alternatively, the fourth and fifth runs in the learning group were the same, indicating the learning processes reaching the performance’s saturation. For the 25 learning group participants, the last epoch of the fifth run was an average of 173.673 ± 19.417 ms. These data indicate that the first-look sequence required approximately 393 ms of transition time, while repeating the same sequence with speed pressure enhanced performance up to approximately 173 ms, demonstrating a 54% improvement.

The difference in the initial transition time between the learning and control groups reflects two factors. First, the difference in instructions is important. For the learning group, the sequence was given before the fMRI scan, whereas for the control group, the sequences were not specified until the scan started. Second, the number of repetitions differed between groups. For example, in the first epoch, the sequence learning group performed a single, pre-determined, five-digit sequence for 30 s, whereas the control group conducted four different sequences for 30 s. We suggest that these factors may affect the initial decline of the transition time.

### Task-based fMRI findings

As represented in Fig. 4, we observed similar spatial patterns of activity in the execution and preparation phases. These areas represent the large-scale functional motor network, necessary for performing sequential motor tasks. The selection of a particular motor sequence is based on inputs from the prefrontal cortex and parietal-temporal regions to the ventral premotor cortex (PMv)^54,55^. The dorsal portion of the IPL (dIPL) is a multimodal sensory association region involved in the initial acquisition and learning of a motor task. The anterior portions of the IPL, PMv, and M1 consist of the fine motor control network, and the PMd is involved in movement selection^55–59^. In addition, preparation-related activity was most prominently associated with enhanced activity in the putamen (Fig. 4B), suggesting that this preparatory activity precedes self-initiated movements^60^. Our findings are consistent with previous results demonstrating preparatory activity in the motor, somatosensory, parietal, and prefrontal cortical regions, basal ganglia, and cerebellum in sequential finger movements^61^.

We observed that preparation-related activity increased linearly in fronto-parietal regions, especially in the right M1 (Fig. 5B). This is consistent with our previous study regarding explicit motor sequence learning, wherein participants needed to internally retrieve whole-sequence information at the preparation phase^11^. Electrophysiological studies in non-human primates have demonstrated an increase in neuronal responses reflecting preparatory activity for movement in the M1 as learning progresses^62^. Thus, the increase in preparation-related activity represents motor learning as an ecphoric process without being confounded by motor execution effects dependent on speed^63–65^ and force^66^. We suggest that the motor learning-related information of the specific sequence was accumulated in the M1 because no such effect was observed in the control group. In addition, an increment of the preparatory activity was highly present in regions that include the SMN and FPN, thereby suggesting that learning-related information is distributed in networks associated with both motor and executive controls.

### MRS findings

First, we found a decrease in Glu concentration within the right M1 in both groups, suggesting that the execution of left-handed movements was not specific to sequence learning. Second, the changes in Glu and GABA were positively correlated, suggesting their dependence on one another and may be because the majority of GABA is formed directly from Glu^27,67^.

In the present study, the GABA concentration was the primary focus because recent studies have suggested that the disinhibition in local circuits is tightly related to learning processes^8^. Glu and GABA MRS measures reflect the local metabolic concentration, and are not specific to synaptic neurotransmitters. Only a small fraction of GABA was found in synaptic vesicles, as opposed to the cytoplasmic pool^27^; however, task execution with learning-related changes should be reflected in the synaptic connection mediated by these neurotransmitters, which are reflected in local metabolic pools. Therefore, we adopted the normalized GABA concentration change (GABA/Glu) and found that the performance change in sequence learning was negatively correlated with the GABA/Glu change (Fig. 3). There were both positive and negative GABA/Glu changes across participants, contrasting previous studies demonstrating GABA reductions in the M1 during motor learning^8,68^. Kolasinski et al.^8^ utilized explicit SRTT, where the engram was found to be localized in the M1 and PMd^10^. Hamano et al.^10^ also demonstrated that the sequential finger tapping learning with speed pressure represented the engram in a network covering the left anterior intraparietal sulcus and IPL. As corticocortical fibers of pyramidal neurons are glutamatergic^69–71^, these findings suggest that between-participant variation in the balance of GABA and Glu reflects the modulatory processes from remote areas, rather than the engram formation localized in the M1.

### Resting-state fMRI findings

We assessed resting-state M1 seed-based functional connectivity changes elicited by motor sequence learning. As shown in Fig. 6, a negative correlation was observed between changes in the GABA/Glu ratio within the M1 and M1 seed-based resting-state functional connectivity changes in the FPN. In contrast, no correlation was found in the SMN. In the learning group, increases in M1-FPN functional connectivity were associated with a decrease in the GABA/Glu ratio in the M1. Since no such relationship was found in the control group, this suggests that GABA/Glu in the M1 represents a remote connection relevant for the learning processes (Fig. 6). The FPN controls coordinated behavior in a rapid, accurate, and flexible goal-driven manner^18^. Therefore, this finding indicates that motor learning driven by cognitive control is associated with local changes in GABA/Glu in the M1, which may indirectly reflect the disinhibitory processes during learning. These findings reflect individual differences in skills, effort, and concentration of self-paced movements because participants were required to execute the task as quickly as possible during learning.

To further investigate the relationship between the M1 and FPN, we assessed the correlations between connectivity changes in the bilateral PFC and PPC and changes in the GABA/Glu ratio of the M1. These correlations were stronger in parietal regions than in frontal regions, suggesting that the GABA/Glu ratio of the M1 is more likely to affect the connectivity with the PPC in the FPN (Fig. 7). This finding is consistent with the notion that the PPC is necessary for early and late learning phases, whereas the PFC is primarily involved in early learning phases^72^. The PFC processes sensory input, motor output, and working memory, whereas the PPC, encompassing the IPL and SPL, processes spatial-sequential components^24,73–76^. Both the M1 and PPC are critical hubs for the late motor sequence learning phase because these areas contribute to the delayed recall of learned motor sequences^77,78^. Therefore, in the later phases of learning, the PPC and M1 are involved in retrieving the learned sequences acquired during the early learning phase. Our results, combined with our previous data, suggest that the M1 integrates the accumulated information processed by the PPC in motor sequence learning.

These findings are consistent with those of Sami et al.^79^, who investigated the consolidation effects of the resting-state network using dual regression independent component analysis (ICA) following implicit and explicit learning, with SRTT. Sami et al. demonstrated the role of the FPN in an explicit learning group, 6 h following the initial acquisition and interpreted this finding as bringing the learned sequence back to declarative awareness. Furthermore, they directly compared explicit and implicit groups at this late state, thereby identifying bilateral activation in both the parietal and premotor regions. They also speculated that this network may represent an engram of the extra procedural learning skill that had developed in the explicit acquisition group^79^. Therefore, we conclude that the M1-centered network with the FPN represents the formation of declarative procedural skills.

### Methodological considerations of MRS

We utilized water data to control the quality of the MRS measurement. We confirmed the water signal linewidth was constant before starting all scans to ensure there was no frequency drift induced by the preceding fMRI. Motion-corrupted scans were already removed before averaging the raw MRS data, and unsuppressed water data could be utilized to estimate absolute concentrations of metabolites^80^. We expressed the concentrations of metabolites relative to tCr (i.e., phosphocreatine [PCr] + creatine [Cr]) as an internal reference, in which the level is explicitly assumed to be stable^27^. We confirmed the stability of tCr by calculating the absolute concentration with water data.

In the present study, we adopted the non-edited Stimulated Echo Acquisition Mode (STEAM). The common MRS method of spectral editing was used to separate the GABA signal from other metabolite signals^81^. Although editing is invaluable for the unequivocal detection of small, obstructed resonances, long echo times (TE) render the quantification of metabolite concentrations susceptible to low SNR and confounding by transverse relaxation times (T2). Although short-TE STEAM is advantageous in this regard, GABA measurements are complicated by overlapping signals^82^. Higher magnetic field strength (≧ 7T) provides higher SNR and spectral dispersion, which would improve the quantification sensitivity and specificity. Thus, the use of ultrahigh field MR systems may relax the need for spectral editing^81^. Importantly, previous studies have shown that GABA could be reliably measured using STEAM at 7T^83,84^.

We utilized 64 averages for STEAM. One previous study^85^ examined the effect of the number of averages on the repeatability of brain metabolite concentrations quantified with STEAM at 7T. The authors suggested that a larger number of averages would be beneficial, but that 32 was acceptable in terms of repeatability. Thus, we concluded that 64 averages would be sufficient for 7T.

### Limitations

The participants recruited in this study were predominantly women, with body weights of 60 kg or less, which contributed to technical challenges in MRS measurements using a single-transmit 7T MR system. First, the B1 transmit field inhomogeneity was enhanced. The suppression of water signals for the measurement of metabolites may have been insufficient depending on the head size, and it was challenging to obtain good spectral quality. Second, adjustments of MRS sequence parameters may have been necessary, involving a lengthening of measurement time to solve the local specific absorption rate limitations partly defined using body weight. Gender differences are known to affect visuo-motor adaptation learning of throwing^86^; given that the participants in our current study were primarily women, the generalizability of the results remain limited, and further studies are warranted to better understand possible gender differences.

## Conclusion

In conclusion, our findings indicate that motor learning driven by cognitive control is associated with local variations in GABA/Glu in the M1 that regulates remote connectivity with the FPN, constituting the M1-centered motor learning network.

## Data Availability

The datasets generated in this study are available from the corresponding author on reasonable request.
